# Supplementary material for: Modeling the effects of routine screening for accidental lab-acquired infections on the risk of potential pandemic pathogen escape from high-biosafety research facilities
Source: Front Bioeng Biotechnol. 2026 Mar 30;14:1714582. doi: 10.3389/fbioe.2026.1714582 (PMC13070908; doi:10.3389/fbioe.2026.1714582)
Supplement: Supplementary file 1 [file DataSheet1.pdf]

APPENDIX:
PARAMETERS, DIFFERENCE EQUATIONS, AND STATE VARIABLES

Modeling the Effects of Routine Screening for Accidental Lab-Acquired Infections on the Risk of Potential Pandemic Pathogen Escape from High-Biosafety Research Facilities

Table 1A: Parameters		
	**Definition**	**Value, Range, or Equation**
**Pinf**	Pinf: the probability of any particular susceptible becoming infected in a given time-step.	$\text{pinfL} = 1 - (1-\tau)\hat{}(\text{infneighISL} + \text{infneighIPL} * \psi + \text{infneighISG} + \text{infneighIPG} * \psi + \text{infneighIAL} * \zeta + \text{infneighIAG} * \zeta + \text{infneighIPISO} * \psi * \iota + \text{infneighISISO} * \iota)$
$\boldsymbol{\tau}$	Tau: probability an infection is transmitted across a given susceptible-infected edge per time step	0.0125 (produces an R0 of 2.0 in absence of testing)
$\boldsymbol{\theta_L}$	ThetaL: testing rate for all LWs per day	Range: 0 to 1 in increments of 1/7
$\boldsymbol{\theta_G}$	ThetaG: testing rate for GPs per day for exposed, pre-symptomatic	0
$\boldsymbol{\omega_G}$	OmegaG: testing rate for GPs per day for infectious, pre-symptomatic	0
$\boldsymbol{\chi_G}$	ChiG: testing rate for GPs per day for infectious, symptomatic [set to ~0 here]	0.01
$\boldsymbol{\sigma}$	Sigma: peak test sensitivity (i.e. during symptomatic phase of infection)	Range: 50% to 100% in increments of 10%
**Isolation delay**	Average delay in days from positive test result to effective isolation	Range: 0 days to 3 days in increments of 1 day
$\boldsymbol{\epsilon_L}$	Epsilon = transition rate from exposed and noninfectious to pre-symptomatic & infectious for both LW and GP	1/5.2=0.19 [1]
$\boldsymbol{\epsilon_{IAL}}$<br>$\boldsymbol{\epsilon_{IAG}}$	EpsilonIAL = transition rate from exposed and noninfectious LW to permanently asymptomatic LW (over course of infection)<br><br>EpsilonIAG = transition rate from exposed and noninfectious GP to permanently asymptomatic GP (over course of infection)	1/5.2=0.0396 so that asymptomatic share is 0.1725.<br>Avg of all four sources: 17.25%<br>8.44% [2]<br>15.6% [3]<br>20% [4]<br>25% [5]
$\boldsymbol{\delta}$	Delta = transition rate from presymptomatic and infectious to symptomatic and infectious	Best estimate from literature is 1/1.3= 0.77 [1]
$\boldsymbol{\gamma_L, \gamma_G, \gamma_{IS}}$	GammaL, gammaG, and gammaIS are the recovery rate per day for symptomatic infectees.	0.1 [6] [7]
$\boldsymbol{\gamma_{IAL}, \gamma_{IAG}}$	GammaIAL and GammaIAG are the recovery rate per day for asymptomatic infectees.	1/14=0.07 [2]

$\boldsymbol{\pi_L}$, $\boldsymbol{\pi_G}$	PiL and PiG: constant isolation rate of symptomatic LWs and GPs (even without testing)	Set to be 20% of recovery rate: 1/10 *0.2= 0.02
$\boldsymbol{\iota}$	Iota= fraction of transmission that happens even while isolated (relative to non-isolated)	0.2 (i.e. 80% transmission reduction from isolation)
$\boldsymbol{\psi}$	Psi= infectiousness offset for pre-symptomatic relative to symptomatic infectees	0.75 [7] Note this estimate draws from multiple studies
$\boldsymbol{\zeta}$	Zeta= ζ= infectiousness offset for asymptomatic relative to symptomatic infectees	0.5 (rough midpoint of previous study findings):<br>1.0 [8]<br>1/3.85=0.26 [9]<br>1/6.9 =0.144 [10]
$\boldsymbol{\mu}$	Mu= test sensitivity offset for exposed but not infectious relative to symptomatic infectees	1% Day 1 Post Infection, 3% Day 2 Post Infection, 13% Day 3 Post Infection, 24% Day 4 Post Infection, 35% Day 5 Post Infection.<br>Average of 15.2% across days 1-5 [11]
$\boldsymbol{\nu}$	Nu= test sensitivity offset for pre-symptomatic relative to symptomatic infectees	48% day before symptom onset [11]

Table 1B: Difference Equations for Model Transitions	
**Variable**	**Difference Equation**
$\frac{dSL}{d(t)}$ SL: Susceptible Lab Workers	-SL*pinfL
$\frac{dSG}{d(t)}$ SG: Susceptible General Population members	-SG*pinfG
$\frac{dENL}{d(t)}$ ENL: Exposed, Not Infectious Lab Workers	SL*pinfL – ((1-ENLtoENISO)*epsilonL)) - 1/(1/(thetaL*sigma*mu)+isolationdelay) - (1-ENLtoENISO)*(1-epsilonL)*epsilonIAL
$\frac{dENG}{d(t)}$ ENG: Exposed, Not Infectious General Population	SG*pinfG – ((1-ENGtoENISO)*epsilonG)) - 1/(1/(thetaG*sigma*mu)+isolationdelay) - (1-ENGtoENISO)*(1-epsilonG)*epsilonIAG
$\frac{dENISO}{d(t)}$ ENISO: Exposed, Not Infectious, and Isolated	1/(1/(thetaL*sigma*mu)+isolationdelay) - 1/(1/(thetaG*sigma*mu)+isolationdelay) – ENISO*epsilonIS) – ENISO*epsilonIS
$\frac{dIPL}{d(t)}$ IPL: Infectious, Pre-symptomatic Lab Workers	(1-ENLtoENISO)*epsilon – (IPL*(1-newIPISOfromIPL)*delta) – (IPL* 1/(1/(thetaL*sigma*nu)+isolationdelay)))
$\frac{dIPG}{d(t)}$ IPG: Infectious, Pre-symptomatic General Population	(1-ENGtoENISO)*epsilonL – (IPG*(1-newIPISOfromIPG)*delta) – (IPG* 1/(1/(omegaG*sigma*nu)+isolationdelay)))
$\frac{dIPISO}{d(t)}$ IPISO: Infectious, Pre-symptomatic, Isolated	(IPL* 1/(1/(thetaL*sigma*nu)+isolationdelay))) + (IPG* 1/(1/(omegaG*sigma*nu)+isolationdelay))) + ENISO*epsilonIS – IPISO*delta
$\frac{dISL}{d(t)}$ ISL: Infectious, Symptomatic Lab Workers	(IPL*(1-newIPISOfromIPL)*delta) – (ISL*(1-newISISOfromISL)*gammaL) - ISL* (1/(1/(thetaL*sigma*(1-piL) +piL)+isolationdelay)))
$\frac{dISG}{d(t)}$ ISG: Infectious, Symptomatic General Population	(IPG*(1-newIPISOfromIPG)*delta) – (ISG*(1-newISISOfromISG)*gammaG) – ISG*(1/(1/(chiG*sigma*(1-piG)+piG) +isolationdelay)))
$\frac{dIAL}{d(t)}$ IAL: Infectious, Asymptomatic Lab Workers	(1-ENLtoENISO)*(1-epsilonL)*epsilonIAL – (IAL*(1-newISISOfromIAL)*gammaIAL) – IAL*thetaL*sigma*0.8
$\frac{dIAG}{d(t)}$ IAG: Infectious Asymptomatic General Population	(1-ENGtoENISO)*(1-epsilonL)*epsilonIAL – IAG*gammaIAG
$\frac{dISISO}{d(t)}$ ISISO: Infectious, Symptomatic, Isolated	(ISL*1/(1/(thetaL*sigma*(1-piL) +piL)+isolationdelay)) + ISG*(1/(1/(chiG*sigma*(1-piG)+piG) +isolationdelay)) + IAL*(thetaL *sigma*0.8) + (IPISO*delta) – ISISO*gammaIS
$\frac{dR}{d(t)}$ Removed	(ISL*(1-newISISOfromISL)*gammaL) + (ISG*(1-newISISOfromISG)* gammaG) + (ISISO*gammaIS) + (IAL*(1-newISISOfromIAL)*gammaIAL) + (IAG*gammaIAG)

Table 2A: State Variables	
**SL**	Susceptible lab worker
**SG**	Susceptible general population member
**ENL**	Exposed but not yet infectious lab workers
**ENG**	Exposed but not yet infectious general population member
**ENISO**	Exposed, not yet-infectious isolated individuals
**IPL**	Infectious, pre-symptomatic lab worker
**IPG**	Infectious, pre-symptomatic general population member
**IPISO**	Infectious, pre-symptomatic isolated person
**ISL**	Infectious, symptomatic lab worker
**ISG**	Infectious, symptomatic general population member
**ISISO**	Infectious, symptomatic or permanently asymptomatic isolated person
**IAL**	Lab worker who is infectious but asymptomatic through whole infection
**IAG**	General population member who is infectious but asymptomatic through whole infection
**R**	People who have progressed through their infections and are either recovered or dead
**InfneighISL**	Number of infected neighbors in the ISL state
**InfneighIPL**	Number of infected neighbors in the IPL state
**InfneighISG**	Number of infected neighbors in the ISG state
**InfneighIPG**	Number of infected neighbors in the IPG state
**InfneighIAL**	Number of infected neighbors in the IAL state
**InfneighIAG**	Number of infected neighbors in the IAG state
**InfneighIPISO**	Number of infected neighbors in the IPISO state
**InfneighISISO**	Number of infected neighbors in the ISISO state
**InfneighISL**	Number of infected neighbors in the ISL state

Table 3A: Odds Ratios for Test Frequency by Average Isolation Delay (From Logistic Model with Interactions for Outbreaks of 50 or More Infections)							
Tests Per Week	0	1	2	3	4	5	6	7
OR with Avg Isolation Delay of 0 Days, with 95% CI	1.00	0.757 (0.749 to 0.764)	0.573 (0.561 to 0.584)	0.434 (0.420 to 0.446)	0.328 (0.315 to 0.341)	0.249 (0.236 to 0.260)	0.188 (0.177 to 0.199)	0.142 (0.132 to 0.152)
OR with Avg Isolation Delay of 1 Day, with 95% CI	1.00	0.782 (0.770 to 0.793)	0.611 (0.593 to 0.629)	0.478 (0.456 to 0.499)	0.374 (0.351 to 0.396)	0.292 (0.271 to 0.314)	0.229 (0.208 to 0.249)	0.179 (0.16 to 0.197)
OR with Avg Isolation Delay of 2 Days, with 95% CI	1.00	0.808 (0.792 to 0.823)	0.653 (0.627 to 0.678)	0.527 (0.496 to 0.558)	0.426 (0.393 to 0.459)	0.344 (0.311 to 0.378)	0.278 (0.246 to 0.311)	0.224 (0.195 to 0.256)
OR with Avg Isolation Delay of 3 Days, with 95% CI	1.00	0.834 (0.814 to 0.854)	0.696 (0.662 to 0.730)	0.581 (0.539 to 0.624)	0.485 (0.438 to 0.533)	0.405 (0.357 to 0.455)	0.338 (0.29 to 0.389)	0.282 (0.236 to 0.333)

Table 4A: Odds Ratios for a 1 Percentage Point Increase in Test Sensitivity by Average Isolation Delay (From Logistic Model with Interactions for Outbreaks of 50 or More Infections)	
OR with Avg Isolation Delay of 0 Days, with 95% CI	0.992 (0.991 to 0.994)
OR with Avg Isolation Delay of 1 Day, with 95% CI	0.993 (0.991 to 0.995)
OR with Avg Isolation Delay of 2 Days, with 95% CI	0.994 (0.992 to 0.997)
OR with Avg Isolation Delay of 3 Days, with 95% CI	0.995 (0.992 to 0.998)

References:

1. Alene M, Yismaw L, Assemie MA, Ketema DB, Gietaneh W, Birhan TY. Serial interval and incubation period of COVID-19: a systematic review and meta-analysis. *BMC Infect Dis*. 2021;21(1):257. doi:10.1186/s12879-021-05950-x

2. Chen C, Zhu C, Yan D, et al. The epidemiological and radiographical characteristics of asymptomatic infections with the novel coronavirus (COVID-19): A systematic review and meta-analysis. *International Journal of Infectious Diseases*. 2021;104:458-464. doi:10.1016/j.ijid.2021.01.017

3. He J, Guo Y, Mao R, Zhang J. Proportion of asymptomatic coronavirus disease 2019: A systematic review and meta-analysis. *J Med Virol*. 2021;93(2):820-830. doi:10.1002/jmv.26326

4. Buitrago-Garcia D, Egli-Gany D, Counotte MJ, et al. Occurrence and transmission potential of asymptomatic and presymptomatic SARS-CoV-2 infections: A living systematic review and meta-analysis. Ford N, ed. *PLoS Med*. 2020;17(9):e1003346. doi:10.1371/journal.pmed.1003346

5. Alene M, Yismaw L, Assemie MA, et al. Magnitude of asymptomatic COVID-19 cases throughout the course of infection: A systematic review and meta-analysis. Kwok KO, ed. *PLoS ONE*. 2021;16(3):e0249090. doi:10.1371/journal.pone.0249090

6. Singanayagam A, Patel M, Charlett A, et al. Duration of infectiousness and correlation with RT-PCR cycle threshold values in cases of COVID-19, England, January to May 2020. *Eurosurveillance*. 2020;25(32). doi:10.2807/1560-7917.ES.2020.25.32.2001483

7. CDC. CDC COVID-19 Pandemic Planning Scenarios. Published online March 19, 2021.

8. Lee S, Kim T, Lee E, et al. Clinical Course and Molecular Viral Shedding Among Asymptomatic and Symptomatic Patients With SARS-CoV-2 Infection in a Community Treatment Center in the Republic of Korea. *JAMA Intern Med*. 2020;180(11):1447. doi:10.1001/jamainternmed.2020.3862

9. Sayampanathan AA, Heng CS, Pin PH, Pang J, Leong TY, Lee VJ. Infectivity of asymptomatic versus symptomatic COVID-19. *The Lancet*. 2021;397(10269):93-94. doi:10.1016/S0140-6736(20)32651-9

10. Chen Y, Li P, Ding Y, et al. Epidemiological feature, viral shedding, and antibody seroconversion among asymptomatic SARS-CoV-2 carriers and symptomatic/presymptomatic COVID-19 patients. *Journal of Infection and Public Health*. 2021;14(7):845-851. doi:10.1016/j.jiph.2021.05.003

11. Zhang Z, Bi Q, Fang S, et al. Insight into the practical performance of RT-PCR testing for SARS-CoV-2 using serological data: a cohort study. *The Lancet Microbe*. 2021;2(2):e79-e87. doi:10.1016/S2666-5247(20)30200-7
